# TURIS Plasma Vaporization – Initial Romanian Experience with a New Technology

**Published:** 2009

**Authors:** B Geavlete, M Drăguţescu, R Mulţescu, D Georgescu, M Jecu, P Geavlete

**Affiliations:** * ”Sf. Ioan” Clinical Emergency Hospital, Department of Urology, Bucharest, Romania

**Keywords:** benign prostatic hyperplasia, transurethral resection in saline – plasma vaporization of the prostate, IPSS, QoL, Qmax, residual volume

## Abstract

Bipolar electrosurgical approach represents an increasingly acknowledged technology in the treatment of benign prostatic hyperplasia (BPH), and a promising alternative to standard transurethral resection of the prostate (TURP). In this study, we aimed to evaluate a new method, transurethral resection in saline – plasma vaporization of the prostate (TURIS-PVP), by determining its efficiency, safety and short-term postoperative results.

In May 2009, TURIS-PVP was performed in 25 cases of BPH. The investigative protocol included digital rectal examination (DRE), prostatic specific antigen (PSA), International Prostate Symptom Score (IPSS), quality of life (QoL) score, maximum flow rate (Qmax) and abdominal ultrasonography assessing prostate volume and post-voiding residual urinary volume (RV). All patients were investigated 1 month after surgery using IPSS, QoL, Qmax and RV.

TURIS-PVP was successfully performed in all cases. The average BPH size was 53 ml, the mean operating time was 28 minutes, the median catheterization time was 24 hours and the mean hospital stay was 48 hours. No patient required blood transfusions or re-catheterization, and there were no significant intra- or postoperative complications. Preoperatively, the mean value of IPSS was 21.4, the mean QoL score was 4, the mean RV was 72 ml and the mean Qmax was 9.7 ml/s. The 1 month follow-up emphasized a mean IPSS of 4, a mean QoL score of 1.4, a mean RV of 14 ml and a mean Qmax of 21.5 ml/s.

TURIS-PVP represents a valuable endoscopic treatment alternative for patients with BPH, with good efficacy, reduced morbidity, satisfactory follow-up parameters and fast postoperative recovery. IPSS, QoL, Qmax and RV measurements showed significant improvements at the 1 month follow-up.

## Introduction

BPH represents an important health problem of the contemporary society and the most common disease in male urological pathology, describing a prevalence of 60% for patients aged 61-70 years old, 70% for those aged 71-80 years old and 80% for those older than 80 years [**1**, **2**].

Various therapeutic modalities have been described for patients with indications for surgery. According to the EAU Guidelines 2009, monopolar TURP is the treatment of choice for prostates sized 30-80 ml [**3**]. 

During the recent years, several alternatives were introduced, aiming to improve the performances of TURP and to reduce its complications, mainly consisting of bleeding, sepsis and TUR syndrome due to fluid absorption. The most widely used procedures at the moment are represented by Holmium laser enucleation, Green Laser photoselective vaporization and bipolar transurethral resection.

Bipolar electrosurgical technology made transurethral electro-vaporization increasingly popular, especially after the development of Gyrus® PlasmaKinetic® Tissue Management System (Gyrus Medical Ltd., Bucks, UK). This technique already proved to be as effective as TURP for bladder outflow obstruction, as it provided good long-term results and implied fewer early complications [**4**].

A new development of this technique, TURIS plasma vaporization of the prostate (PVP), using the Olympus UES-40 Surgmaster generator (Olympus, Hamburg, Germany) and the “mushroom” vapo-resection electrode, have been recently introduced in clinical practice. 

In Romania, TURIS-PVP was performed as a national premiere in “Sf. Ioan” Clinical Emergency Hospital, Department of Urology, in May 2009. In this study, we aimed to evaluate the efficiency, safety and short-term postoperative results of this new procedure.

## Materials and Methods

In May 2009, TURIS-PVP was performed in 25 men with a mean age of 64 years old (range 53 to 81), diagnosed with BPH and severe lower urinary tract symptoms (LUTS). The informed consent signed by all patients was included in the study. 

**Fig. 1:**
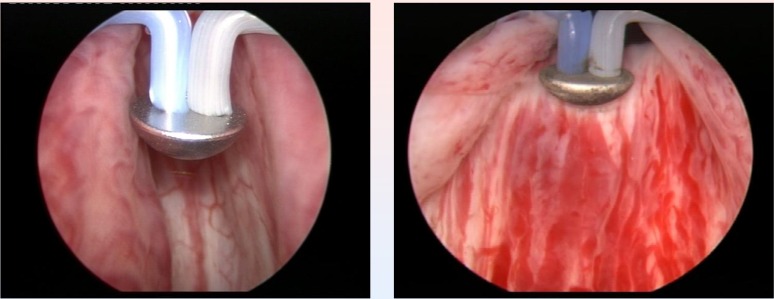
Preoperative BPH images

A standard investigative protocol which included general clinical examination with digital rectal examination (DRE), blood tests, PSA, urine culture, IPSS and QoL score evaluation, uroflowmetry (evaluating Qmax) and abdominal ultrasonography were applied in all cases (**Fig. 1**).

All patients with abnormal DRE and/or increased PSA were excluded from the study.

**Fig. 2:**
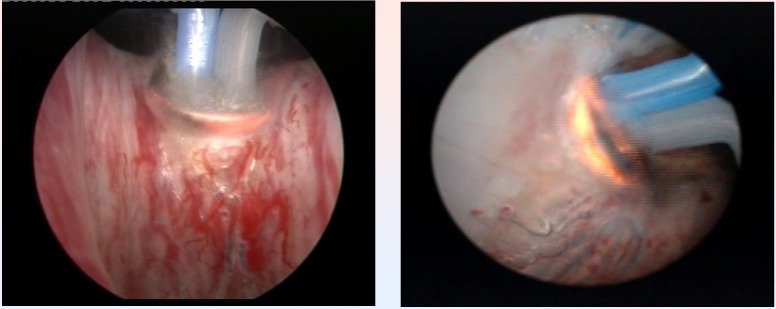
Initial aspects at the beginning of the procedure

The Olympus SurgMaster UES-40 bipolar generator and the special “mushroom” type vapo-resection electrode were used in all cases (**Fig. 2**).

**Fig. 3:**
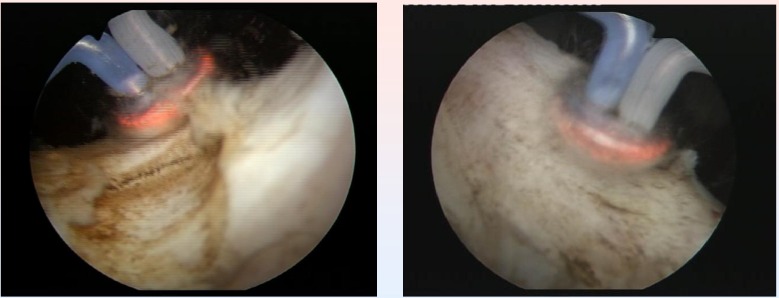
Bladder neck BPH tissue vaporization

The endoscopic procedures were performed under spinal anesthesia and by using saline continuous flow irrigation (**Fig. 3**).

**Fig. 4:**
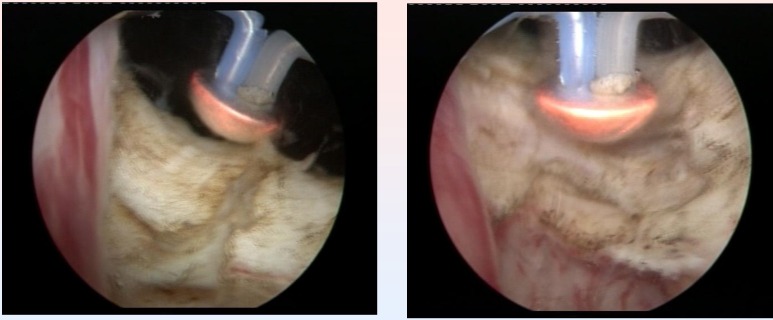
Median lobe plasma vaporization

From the point of view of the surgical technique, the spherical shaped new type of electrode displaying a plasma corona on its surface was gradually moved in direct contact with the BPH tissue (“hovering” technique), thus producing a virtually blood-less vaporization (**Fig. 4**).

Several prostatic fragments were resected, eventually from any suspicious looking tissue area, thus providing substantial specimens for the pathological analysis, in order to confirm the benign nature of the lesion.

Coagulation of any hemorrhagic sources was practically concomitant with the vaporization, while larger vessels hemostasis was achieved by reducing the power of the generator. This parameter was set at 280-320 W for vaporization, varying with tissue consistency, and at 120-140 W for coagulation. 

In all cases, a Foley catheter was placed at the end of the procedure.

The one month postoperative follow-up assessed the IPSS, QoL, Qmax and RV in all patients, and compared them with the preoperative data.

## Results

**Fig. 5:**
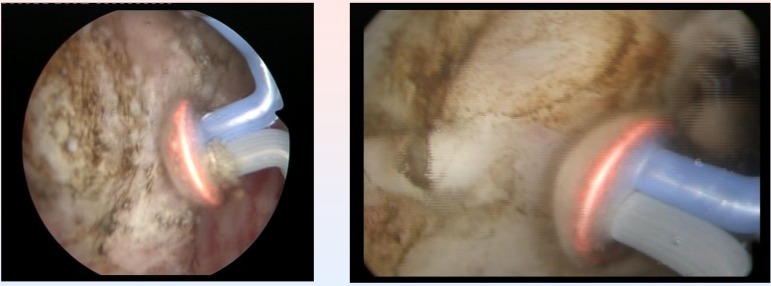
Right prostatic lobe vaporization with the „mushroom” electrode

TURIS-PVP was successfully performed in all cases (**Fig. 5**).

**Fig. 6:**
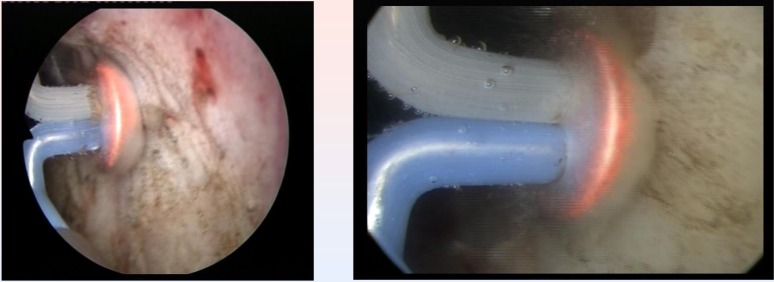
Left prostatic lobe vaporized by the plasma corona on the surface of the hemispherical shape “mushroom” type electrode

The average BPH size was 53 ml (range 32-78 ml). The mean operating time was 28 minutes (range 20-47 minutes) (**Fig. 6**).

The urethral catheter was removed after a median period of 24 hours (range 12-48 hours), and the medium hospital stay was 48 hours (range 36-72 hours). The pre- and postoperative hemoglobin level was 14.6 g/dl and 14.1 g/dl, respectively. 

**Fig. 7:**
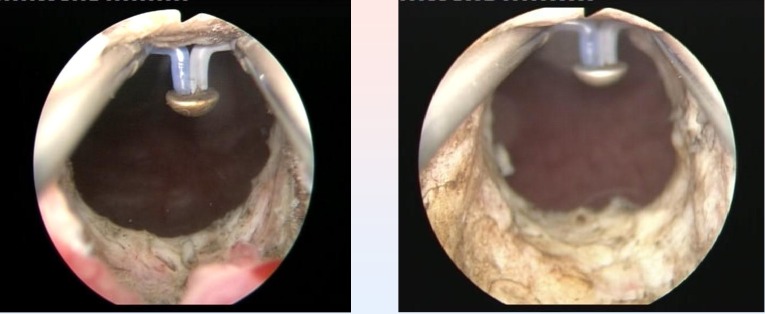
Postoperative images of large passage in the prostatic fossa, without obstruction

No patient required blood transfusions or re-catheterization, and there were no major intra- or postoperative complications. There were no cases of urinary tract infection or sepsis, acute urinary retention, prostatic capsule perforation, profound thermal lesions, significant bleeding or clot retention (**Fig. 7**). 

One patient presented minor postoperative hematuria and another one, moderate dysuria after catheter removal. Both cases were treated conservatively, with no further complications. 

The preoperative patients’ assessment emphasized the following data: the mean value of IPSS was 21.4 (range 15-27), the mean QoL score was 4 (range 3-5), the mean RV was 72 ml (range 0-215 ml) and the mean Qmax was 9.7 ml/s (range 5.2-12.3 ml/s).

At the one month follow-up evaluation, IPSS, QoL, Qmax and RV measurements showed significant improvements, as following: the IPSS was 4 (range 2-7), the QoL score was 1.4 (range 1-2), the RV was 14 ml (range 0-30 ml) and the mean Qmax was 21.5 ml/s (range 15-24.5 ml/s). 

No acute urinary retention, urinary incontinence or significant hematuria occurred during the follow-up period. 

## Commentaries

Despite the fact that conventional monopolar TURP remains the first line treatment option in prostates of 30-80 ml, plasma energy in a saline environment is mentioned as a viable alternative in clinical trials [**3**]. 

**Fig. 8:**
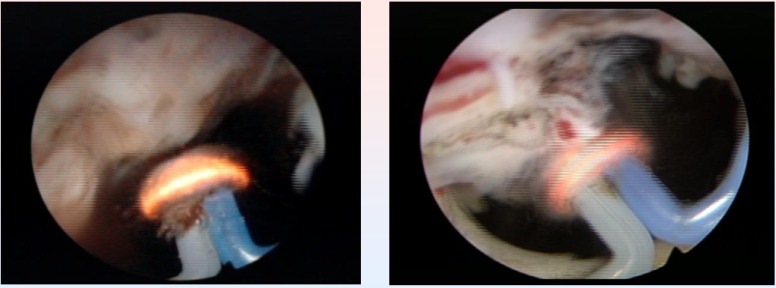
Plasma vaporization of the anterior region of the prostate

The basis of TURIS-PVP is represented by the ability of the UES-40 bipolar electrosurgical generator to produce a plasma corona on the surface of the spherical shaped “mushroom” electrode type. Plasma vaporization occurs by direct gentle contact with the tissue surface, and performs concomitant hemostasis (**Fig. 8**).

**Fig. 9:**
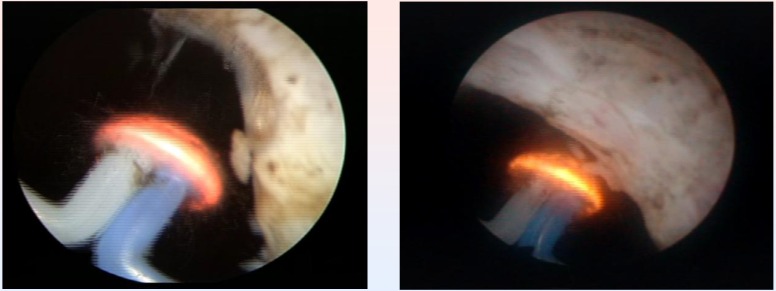
Last remaining details of a TURIS-PVP

Technically speaking, the endoscopic armamentarium proved easy to use, and the general learning curve of the procedure, remarkably short (increased efficiency after the first 5 interventions). During the entire operating time, visibility remained excellent, due to the lack of bleeding and to the high quality of the endoscopic images (**Fig. 9**).

**Fig. 10:**
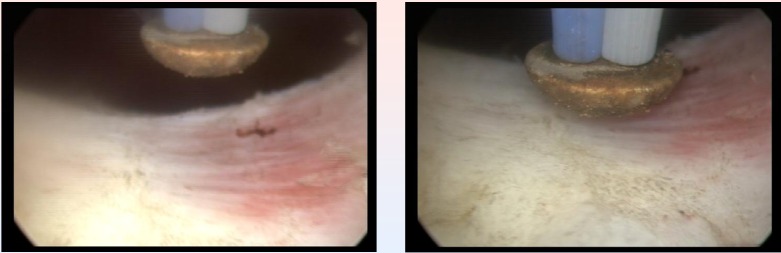
Smooth surface of the prostatic capsule after plasma vaporization

Subjectively, this type of vaporization did not alter the visual characteristics of the tissues, enabling the surgeon to differentiate between the adenomatous tissue, the muscular fibers of the prostatic capsula and the anatomical boundaries of the operating area with increased accuracy. At the end of the procedure, the vaporization area emphasized a remarkably smooth surface and sharp margins, with no irregularities or debris (**Fig. 10**).

The power of the generator was adapted to tissue characteristics and consistency, providing the surgeon additional technical flexibility: 320 W for fibrous tissue, 280-290 W for average BPH tissue, 240 W for remaining BPH small fragments close to the capsule or apex and 120-140 W for coagulation.

While comparing the results of this study with those determined after plasma vaporization using the PlasmaKinetic Tissue Management System, we can underline superior postoperative results for TURIS-PVP at almost every chapter of evaluation: IPSS 4 versus 5, QoL 1.4 versus 1.5, RV 14 ml versus 19.5 ml and Qmax 21.5 ml/s versus 18 ml/s [**5**]. 

Patients included in the study groups presented similar baseline characteristics. The median prostate volume was higher in our study (53 ml versus 42 ml), the operative time was shorter (28 minutes versus 55 minutes), and the catheterization time and hospital stay were also reduced (24 versus 72 hours and 48 versus 72 hours, respectively) [**5**]. Although our study group was rather small, early results predicted significant progresses in terms of short-term outcome for TURIS-PVP in comparison to its predecessor.

It is also imperative to make a comparison between the previous treatment and the “gold standard” endoscopic treatment in BPH, the monopolar TURP.

According to an extensive study, voiding parameters in patients with initially similar obstructive profile and prostate volume emphasized a Qmax of 21.6 ml/s and a RV of 31.1 ml. The mean operating time was 52.4 minutes for an average prostate volume of 44.5 ml. The mean hospital stay for TURP patients was 8 days [**6**]. From this perspective as well, TURIS-PVP seems to bring quite an improvement, as the mean operating time in our study was 28 minutes for an average prostate volume of 53 ml, with a mean hospital stay of 2 days. The average IPSS decrease for TURP reported in the literature (from 18.8 to 7.2) was inferior to that of TURIS-PVP (from 21.4 to 4) [**7**]. The average catheterization time was significantly shorter for TURIS-PVP in comparison to the TURP literature data (24 versus 115 hours) [**8**]. 

It is important to mention that some stages of the conventional procedure are significantly reduced (hemostasis becomes practically concomitant with the actual vaporization, the few resected tissue fragments are quickly evacuated), so the actual vaporization basically occupies the great majority of the operating time, thus increasing the efficiency of the procedure in comparison to TURP.

The most important “weak point” of TURP, the still significant complications’ rate, was described as following: 2.9% transfusion rate, 1.4% TUR syndrome rate, 3.6% UTI rate, 5.8% failure rate and 0.1% mortality rate [**6**]. The fact that none of these complications occurred in our series remains significant, regardless of the small number of cases included in the study.

Due to the rather resembling type of technique, it may be useful to make a comparison between TURIS-PVP and a strongly emerging and much popular procedure, potassium-titanyl-phosphate (KTP) photoselective laser vaporization prostatectomy. In all respects of efficacy and short-term postoperative outcomes, TURIS-PVP seems to be superior (mean prostate volume 53 ml versus 43 ml, mean operative time 28 minutes versus 53 minutes, mean catheter removal time 24 hours versus 33.6 hours, mean hospital stay 48 hours versus 86.4 hours, mean IPSS 4 versus 11.65, mean RV 14 ml versus 23 ml and mean Qmax 21.5 ml/s versus 18.6 ml/s) [**9**].

From the cost-effectiveness point of view, the advantages of TURIS-PVP are quite obvious: both the bipolar generator and the “mushroom” type vapo-resection electrode are about 10 times cheaper than the KTP Laser device and laser fiber, respectively. Both the “mushroom” electrode and the laser fiber are designed for single usage. Moreover, the possibility of tissue samples’ resection during TURIS-PVP provides the patients with a chance for occult prostate cancer pathological diagnostic, an advantage from which KTP laser patients do not benefit from.

The above mentioned data is summarized in **Table 1** and **Table 2** in order to make an adequate comparison between these 4 therapeutic approaches.

**Table 1:** Preoperative parameters

Parameters	TURIS-PVP	PlasmaKinetic	TURP	KTP
IPSS	21.4	22	20.5	19.7
Prostatic vol.	53 ml	42 ml	44.5 ml	43 ml
RV	72 ml	54 ml	180 ml	88 ml
Qmax	9.7 ml/s	8.5 ml/s	10.4 ml/s	13 ml/s

**Table 2:** Surgical and postoperative features

Features	TURIS-PVP	PlasmaKinetic	TURP	KTP
Operating time	28 minutes	55 minutes	52.4 min	53 min
Catheter time	24 hours	72 hours	115 hours	33.6 hours
Hospital stay	48 hours	72 hours	192 hours	86.4 hours
Postop. IPSS	4	5	7.2	11,65
Postop. RV	14 ml	19.5 ml	31.1 ml	23 ml
Postop. Qmax	21.5 ml/s	18 ml/s	21.6 ml/s	18.6 ml/s

Our initial results seem to emphasize a significant superiority of TURIS-PVP over other treatment alternatives, both from the perspective of surgical efficiency and from the point of view of short-term results, under the circumstances of similar preoperative patients’ characteristics.

The long-term follow-up of patients treated by PlasmaKinetic vaporization already demonstrated good stable results: IPSS improvement from 21 to 7.1 and 7.6 at 24 and 36 months, respectively. Qmax increased at 12.5 and 14.4 ml/s at 2 and 3 years, respectively [**10**]. Similar studies need to clarify the long-term results of TURIS-PVP.

## Conclusions

TURIS-PVP represents a valuable endoscopic treatment alternative for BPH patients, with good efficacy, reduced morbidity, satisfactory follow-up parameters and fast postoperative recovery. 

The short hospital stay, reduced the rate of complications, virtually inexistent intra- and postoperative bleeding, short period of catheterization and satisfactory follow-up features (IPSS, QoL, Qmax and RV) are strong arguments in favor of this new technology. 

The cost-effectiveness of this procedure seems superior to that of other minimally invasive alternatives, and the short-term results seem quite similar to those of conventional TURP. 

Future studies, including large number of cases, are due to establish the long-term advantages and general viability of the method. 
